# Supplementary material for: The short conserved region-2 of LARP4 interacts with ribosome-associated RACK1 and promotes translation
Source: bioRxiv. 2024 Nov 1:2024.11.01.621267. Preprint. [Version 1] doi: 10.1101/2024.11.01.621267 (PMC11565960; doi:10.1101/2024.11.01.621267)

bioRxiv preprint doi: https://doi.org/10.1101/2024.11.01.621267; this version posted November 1, 2024. The copyright holder for this preprint (which was not certified by peer review) is the author/funder. This article is a US Government work. It is not subject to copyright under 17 USC 105 and is also made available for use under a CC0 license.

SUPPLEMENTARY MATERIAL FOR

The short conserved region-2 of LARP4 interacts with ribosome-associated RACK1 and promotes translation

Amitabh Ranjan[1], Sandy Mattijssen[1], Nithin Charlly[1], Isabel Cruz Gallardo[2,#], Leah F. Pitman[1,3,+], Jennifer C. Coleman[2,°], Maria R. Conte[2,^], and Richard J. Maraia[1*]

[1]Section on Molecular and Cellular Biology, *Eunice Kennedy Shriver* National Institute of Child Health and Human Development, National Institutes of Health, Bethesda, MD

[2]Randall Centre for Cell and Molecular Biophysics, King's College London, London SE1 1UL, UK

[3]Messenger RNA Regulation and Decay Section, Center for Cancer Research, National Cancer Institute, Frederick, MD, United States.

*Corresponding author: maraiar@mail.nih.gov
^Co-corresponding author: sasi.conte@kcl.ac.uk

[#]Present address: Department of Chemistry, King's College London, Britannia House, 7 Trinity St, London SE1 1DB, UK.

[+]Present address: MRC Laboratory of Molecular Biology, Francis Crick Avenue, Cambridge Biomedical Campus, Cambridge CB2 0QH UK

[°]Present address: Cancer Research UK Cambridge Institute, Li Ka Shing Centre, University of Cambridge, CB2 0RE, UK

SUPPLEMENTARY FIGURE LEGENDS

Supp Figure S1: A-C) Yeast two hybrid domain mapping growth results according to plating layout design in panel **D)** with component constituents in panel **E)**. **A-C** are as in Figure 1, spotted cells grown from three isolated colonies on agar plates containing the selective media listed above. SD –Leu,-Trp selects for presence of the bait and prey plasmids respectively; SD –Leu,-Trp,-His additionally tests for interaction via production of the reporter gene product His3; and SD –Leu,-Trp,-His, +10 mM 3AT blocks function of the reporter product.

Supp Figure S2: A) Multiple sequence alignment (MSA) of LARP4 and LARP4B numbered above corresponding to conserved region 2 (CR2) of human LARP4, followed by bracket indicating a proline-rich region; asterisks above the sequence denote reciprocal/switched Leu and Pro in LARP4 and 4B.

Supp Figure S3: Five superimposed LARP4-RACK1 models predicted by AlphaFold-Multimer (AFM) corresponding to Fig 2E. The protein sequences input were LARP4(401-724) and RACK1 full length. **A)** LARP4 regions were rendered invisible except for those extending locally from CR1 and CR2 sequence motifs, the boundaries of which are indicated by dashed lines. RACK1 is black. ChimeraX Matchmaker analysis of the five AFM predictions revealed good agreement for RACK1 (RMSD across the 317 residue atom pairs whereas LARP4(401-724) agreement was limited to the CR1 and CR2 sequence regions. **B)** Video: see separate MP4 file. The five superimposed models predicted by AFM; as in A but with the views of LARP4 amino acids 401-724 visible.

Supp Fig S4: A) Five superimposed LARP4B-RACK1 models predicted by AFM corresponding to Fig 2J. The protein sequences input were LARP4B(433-738) and RACK1 full length. LARP4B regions were rendered invisible except for those extending locally from CR1 (green) and CR2 (pink) sequence motifs, RACK1 is black. ChimeraX Matchmaker analysis of the five predictions revealed good agreement for RACK1 (RMSD across the 317 residue atom pairs) whereas LARP4B(433-738) agreement was limited to the CR1 and CR2 sequence regions. **B)** Video: see separate MP4 file. The five superimposed AFM-predicted models as in A but with LARP4B amino acids 433-738 visible. **C-D)** Results of AFM random seed iterative model predictions. **C)** Protein sequence inputs were: RACK1 and only the 17-mer CR2 sequence, LARP4B(644-660). The figure shows converged predicted models (124 out of 125 models). **D)** Protein sequence inputs were: RACK1 and the 13-mer CR1 within a longer sequence, LARP4B(513-555); Left: RACK1 in ribbon view in orientation as in Fig 2I. Middle and right: rotated views.

bioRxiv preprint doi: https://doi.org/10.1101/2024.11.01.621267; this version posted November 1, 2024. The copyright holder for this preprint (which was not certified by peer review) is the author/funder. This article is a US Government work. It is not subject to copyright under 17 USC 105 and is also made available for use under a CC0 license.

Supp Figure S5: A) Northern blot oligo probes specific for β-Globin or β-Globin ARE reporter mRNAs corresponding to figures 3C and 4I. Schematic representation of the mature β-globin mRNA and β-globin-ARE mRNA reporters. The gene-specific probes used for the blots in the main figures are indicated by the 2 or 3 color bars below the cartoons. The β-globin-CDS probe indicated above the cartoons was used for the blots in this supplementary figure, for demonstrative purposes, as annotated. A single blot loaded with four lanes of β-glo mRNA on the left side and four lanes of β-globin-ARE mRNA on the right side, revealed all lanes by the β-globin-CDS probe. After stripping of the probe and subsequent reprobing, each gene-specific probe detected its target mRNA but not the other. **B)** Schematic representation of the plasmid reporter genes used in transient transfection experiments. Blue regions represent coding sequences; the stop codon is indicated by a thick vertical line. The β-globin and β-globin-ARE reporters differ only by the presence/absence of the 38 nt ARE in the 3'-UTR.

Supp Figure S6: Replicate polysome profile data supporting figure 4, and quantitative analysis of β-globin-ARE mRNA distribution in polysome and pre-polysome fractions from three biological replicates. The layout is like figure 4. A) Immunoblot of input lysates from samples with transfected constructs indicated above the lanes, EV (empty vector), L4-WT and L4-R1 probed with anti-FLAG Ab. **B)** Polysome profiles of the lysates run in parallel, representing EV, L4-WT and L4-R1 under which are an Ethidium Bromide-stained agarose gel, an immunoblot processed as indicated to the left (**C-E**), and a northern blot probed for various mRNAs (**F-L**), stacked in columns i-iii. **M-O)** Triplicate data set based on quantification of the distribution of β-glo-ARE mRNA among polysome profile fractions of three independent sucrose gradient sedimentations as in Fig 4, Supp Fig S5 and another. **M:** Graph of % β-glo-ARE mRNA in polysome vs. pre-polysome fractions; collective % in fractions 9-13 and in fractions 1-7 as indicated by bar patterns for EV, LARP4-WT and LARP4-R1. P value ***$p<0.001$ determined by two-tailed student's T test; ns = non-significant. **N:** Triplicate β-glo-ARE mRNA polysome profile distribution in which amounts in each fraction set was plotted as bars. **O:** Same as in N, plotted as lines.

Supp Figure S7: Analysis of LARP4 cosedimentation with ribosomes by S100 sucrose cushion; replicate data related to figure 4F. Here are the three replicate sucrose cushion S100 sedimentation analysis, each on an immunoblot processed for detection of FLAG-L4, RPS6 and RPL9. The inputs (in), supernatants (S), and pellets (P) for the lysates from EV, LARP4-WT and LARP4-R1 samples are as indicated.

Supp Figure S8: Co-IP of PABP with FLAG-LARP4; replicate data sets related to figure 4H. A) Panels i, ii, iii and iv are immunoblot results of a single IP experiment in which all samples were processed side-by-side but too numerous to fit on one gel. The LARP4 mutated proteins indicated above the lanes were expressed as FLAG-tagged by transfection. Extracts were prepared and IPed using anti-FLAG Ab. The blots in each panel show input extracts, IP products and supernatants, as indicated, processed for detection using anti-PABP (upper) and anti-FLAG (lower). **B)** Independent experiment in which IP samples were on one blot and inputs on another; FLAG-La was a negative control. **C)** An independent experiment in which only L4-WT, L4-R1 and La protein were compared for co-IP of PABP. The anti-FLAG and anti-PABP are distinguished by color; FLAG-La was a negative control.

Supp Figure S9: LARP4 activity increases translation efficiency of nanoLuciferase (nLuc) reporter mRNA. A-E) As in figure 5, 48 h after cotransfection of the nLucP-ARE reporter plasmid and the LARP4-CS (codon swap) expression plasmid, cells were harvested and half processed for protein and half for RNA. To each of the transfection reaction mixtures containing equal amounts of the nLucP-ARE reporter plasmid, was added the different amounts of LARP4-CS plasmid in μg indicated above the lanes (0 = empty plasmid). **A-C:** Analysis of LARP4-CS expression by immunoblot developed using anti-FLAG (A), anti-actin (B), and the results were quantified and graphed (C). **D:** Northern blot analysis of nLucP-ARE mRNA expression in the same samples as in A-B as indicated above the lanes. The nLucP-ARE RNA reactive with the probe was quantified and indicated below the lanes. The double ended arrow at the left annotated with long and short refers to poly(A) length differences characteristic of LARP4 activity. A dashed line rectangle helps discern the lower and upper boundaries of that reflect poly(A) lengths that contribute to the mobility shift[1-3]. **E:** The upper graph plots nLucP luciferase activity in relative light units (RLU)/μg extract (Y-axis) versus LARP4-CS protein levels (X-axis). The R^2 value derived from a linear fit trendline is shown as a dashed line. The lower graph plots TE, (nLucP luciferase activity/μg extract)/(nLucP-ARE mRNA/μg extract) on the Y-axis versus LARP4-WT levels (X-axis)**.** The R^2 value derived from a linear fit trendline is shown as a dashed line.

F-I) Analysis of four nLuc reporters integrated in Flp-In HEK293 cells, and responses to LARP4-WT and LARP4-R1. **F:** Diagram showing experimental schema as in Figure 5. **G:** Northern blot analysis. The four panels contain RNAs from cells treated as indicated above the lanes, from the Flp-In T-Rex line containing the single copy reporter: nLuc, nLucP, nLuc-ARE and nLucP-ARE. All samples were processed side-by-side; the two left and two right panels

bioRxiv preprint doi: https://doi.org/10.1101/2024.11.01.621267; this version posted November 1, 2024. The copyright holder for this preprint (which was not certified by peer review) is the author/funder. This article is a US Government work. It is not subject to copyright under 17 USC 105 and is also made available for use under a CC0 license.

were run on different gels and transferred to different blots which were hybridized with the same antisense nLuc probe, washed together, and exposed on the same phosphorImager screen. Quantification of the nLuc signal/GAPDH is shown below the lanes. **H:** Immunoblot analysis of the FLAG-LARP4 levels as indicated. **I:** Nanoluciferase activities in the protein extracts corresponding to the samples in G; note different scales on Y-axes. **J:** Translation efficiency (TE). The nanoluciferase activity data from I was normalized by the corresponding nLuc-reporter mRNA and plotted.

Supp Figure S10: **A)** Views of RACK1 as it resides on an existing structure of the human 80S ribosome (PBD: 6Z6M[4 see 5]. The upper structure was oriented such that RACK1 is central and facing the viewer; it and the three native ribosomal proteins in close contact, RPS3, RPS17, and RPS6, were rendered in surface view as indicated. For the bottom panel, all elements in PBD 6Z6M other than the four shown above in surface view were made invisible, and features of RACK1 were annotated (see Fig 2E). **B)** AlphaFold-Multimer was used in standard mode to predict a model assembled from RACK1, and sequences representing LARP4, RPS3, RPS17, and RPS6 (Methods), sequence coverage for each component was >1000. LARP4 residues were rendered invisible except for those extending locally from the CR1 and CR2 sequences. The best model is shown. AFM produced a predicted assembly similar in general orientation to that in the lower panel of A. In B, the CR2 and CR1 motifs are engaged in much the same way as for RACK1 alone.

Supp Figure S11: **β-glo-ARE mRNA stabilization in LARP4-KO cells is rescued differentially by LARP4-WT** and **LARP4-RACK1 mutants. A)** Immunoblot of extract protein from HEK293 LARP4-KO cells transfected with the LARP4 (L4) constructs above the lanes; EV: empty vector (see text). **B)** Northern blot shows simultaneous responses of β-glo-ARE and GFP mRNAs in the same pool of transfected cells carrying a different LARP4 mutant. **C)** Quantitation of northern blot signals of biological duplicate experiments. Transcript levels were normalized by GAPDH mRNA.

REFERENCES

1. Iben, J.R., Li, T., Mattijssen, S. and Maraia, R.J. (2024) Single-Molecule Poly(A) Tail Sequencing (SM-PATseq) Using the PacBio Platform. *Methods Mol Biol*, **2723**, 285-301.
2. Kozlov, G., Mattijssen, S., Jiang, J., Nyandwi, S., Sprules, T., Iben, J.R., Coon, S.L., Gaidamakov, S.A., Noronha, A.M., Wilds, C.J. *et al.* (2022) Structural basis of 3'-end poly(A) RNA recognition by LARP1. *Nucleic Acids Research* **50**, 9534-9547.
3. Mattijssen, S., Iben, J.R., Li, T., Coon, S.L. and Maraia, R.J. (2020) Single molecule poly(A) tail-seq shows LARP4 opposes deadenylation throughout mRNA lifespan with most impact on short tails. *Elife*, **9**, 9:e59186.
4. Wells, J.N., Buschauer, R., Mackens-Kiani, T., Best, K., Kratzat, H., Berninghausen, O., Becker, T., Gilbert, W., Cheng, J. and Beckmann, R. (2020) Structure and function of yeast Lso2 and human CCDC124 bound to hibernating ribosomes. *PLoS Biol*, **18**, e3000780.
5. Rollins, M.G., Shasmal, M., Meade, N., Astar, H., Shen, P.S. and Walsh, D. (2021) Negative charge in the RACK1 loop broadens the translational capacity of the human ribosome. *Cell Rep*, **36**, 109663.

bioRxiv preprint doi: https://doi.org/10.1101/2024.11.01.621267; this version posted November 1, 2024. The copyright holder for this preprint (which was not certified by peer review) is the author/funder. This article is a US Government work. It is not subject to copyright under 17 USC 105 and is also made available for use under a CC0 license.

Supp Figure S1 Ranjan et al.

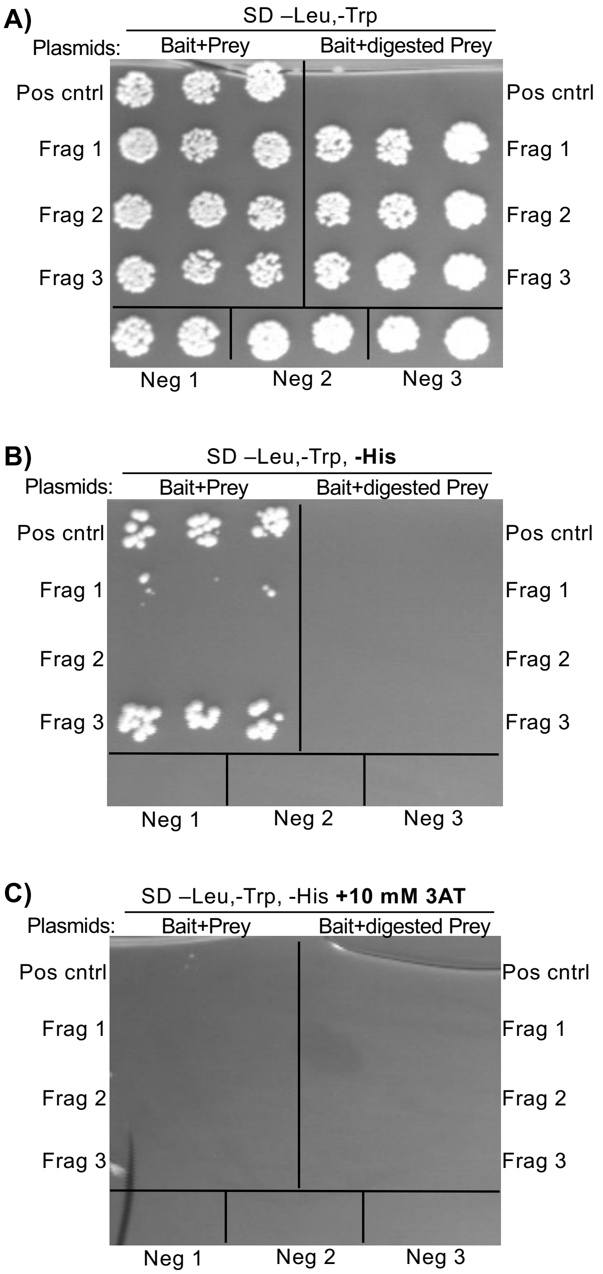

D)

Tested interactions and controls Fragments 1 to 3

Pos cntrl	1	1	1				Pos cntrl
Frag 1	5	5	5	6	6	6	Frag 1
Frag 2	7	7	7	8	8	8	Frag2
Frag 3	9	9	9	10	10	10	Frag 3
	2	2	3	3	4	4	
	Neg 1		Neg 2		Neg 3		

E)

	**Test description**	**Prey construct**	**Bait construct**
1	Positive control	GNB2L1	LARP4, initial bait
2	Neg control 1 (Neg1)	pP7	LARP4, initial bait
3	Neg control 2 (Neg2)	GNB2L1	pB27
4	Neg control 3 (Neg3)	GNB2L1	pB27 linearized
5	Interaction Frag 1	GNB2L1	pB27 linearized +Frag 1
6	Neg control Frag 1	pP7	pB27 linearized +Frag 1
7	Interaction Frag 2	GNB2L1	pB27 linearized +Frag 2
8	Neg control Frag 2	pP7	pB27 linearized +Frag 2
9	Interaction Frag 3	GNB2L1	pB27 linearized +Frag 3
10	Neg control Frag 3	pP7	pB27 linearized +Frag 3

Supp Figure S2 Ranjan et al.

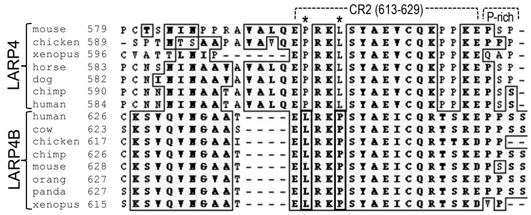

Supp Figure S3, Ranjan et al.

A) Five superimposed AF-M predicted models from which the LARP4-RACK1 best model in Fig 2E were derived; only immediate sequences flanking the CR2 and CR1 of the LARP4 chain are shown.

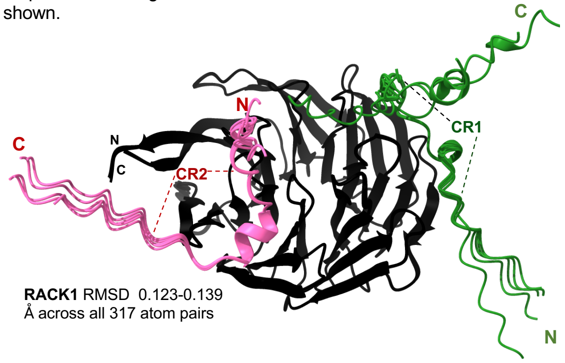

B) Supp Video Figure. Five superimposed AF-M predicted models from which the best model in Fig 2E were derived

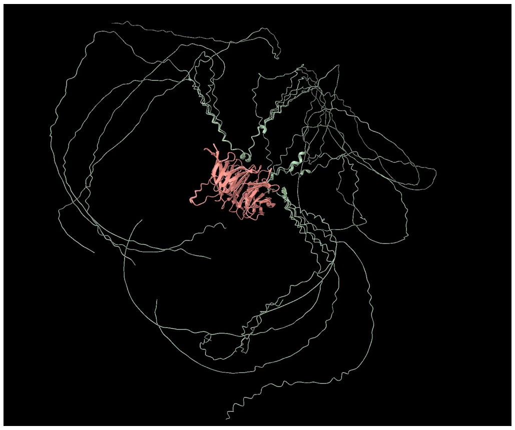

Supp Figure S4, Ranjan et al.

A) Five superimposed AF-M predicted models from which the LARP4B-RACK1 best model in Fig 2I were derived; only immediate sequences flanking the CR2 and CR1 of the LARP4 chain are shown.

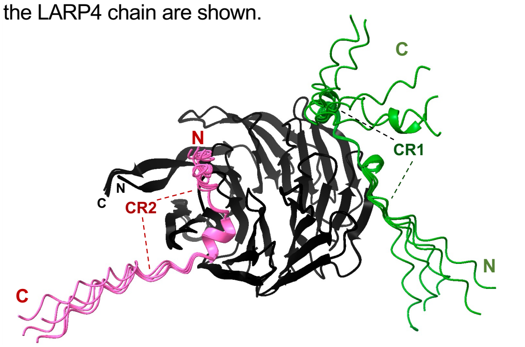

RACK1 RMSD 0.122-0.184Å
across all 317 atom pairs

B) Supp Video Figure. Five superimposed AF-M predicted models from which the LARP4B-RACK1 best model in Fig 2I were derived

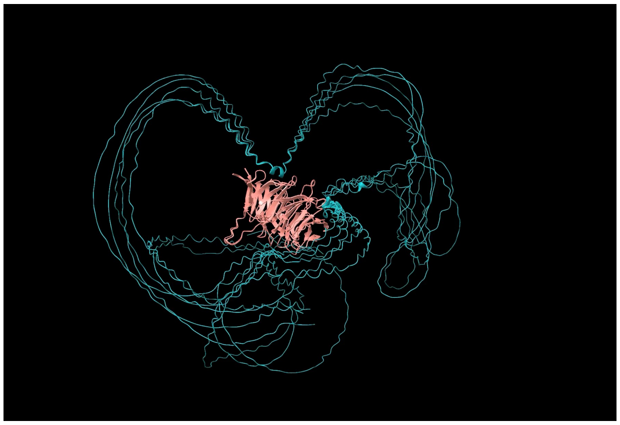

C) Alpha-Fold-Multimer random seed iterative structure prediction models of RACK1 and the 17-mer LARP4B CR2; 124 of 125 converged predicted structures are shown

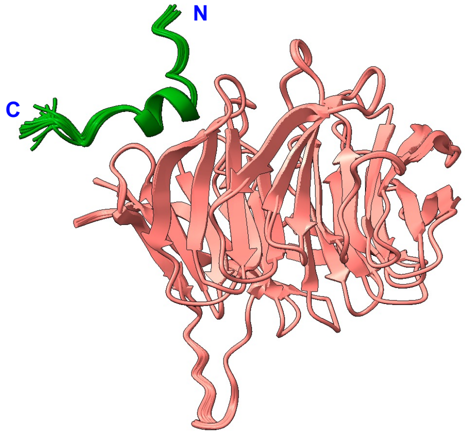

D) Alpha-Fold-Multimer random seed iterative structure prediction models of RACK1 and the LARP4B CR1; 24 of 25 converged predicted structures are shown.

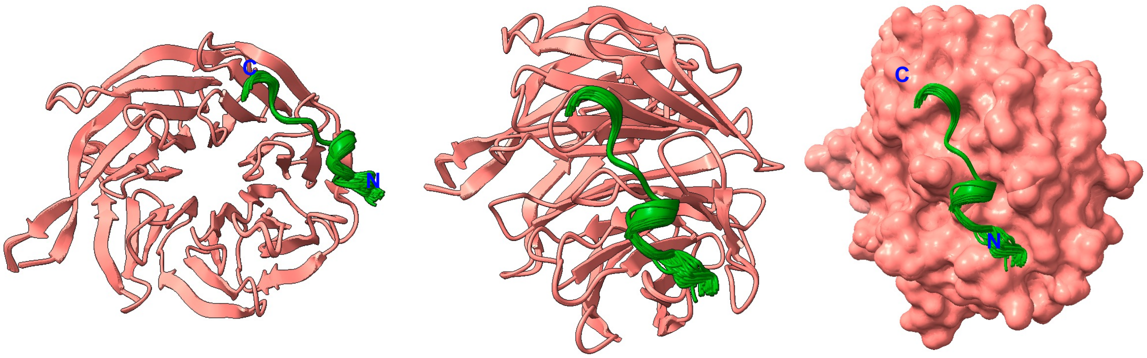

Supp Figure S5, Ranjan et al.

A) Proof of specificity principle experiment uses northern blot probes specific for either β-globin or β-globin-ARE reporter mRNAs, and a β-globin-CDS probe that recognizes both.

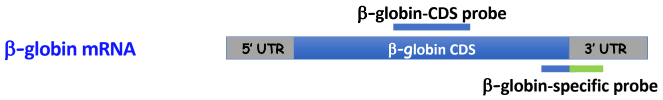

β-globin-specific probe can detect β-globin-mRNA, but not β-globin-ARE mRNA.

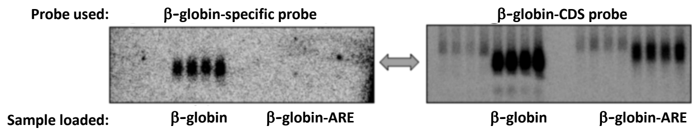

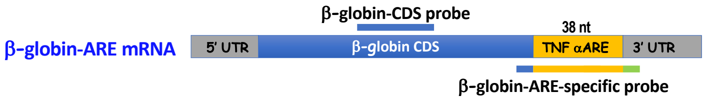

β-globin-ARE-specific probe can detect β-globin-ARE mRNA, but not β-globin.

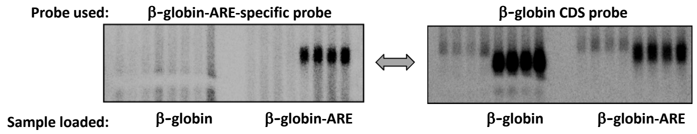

B)

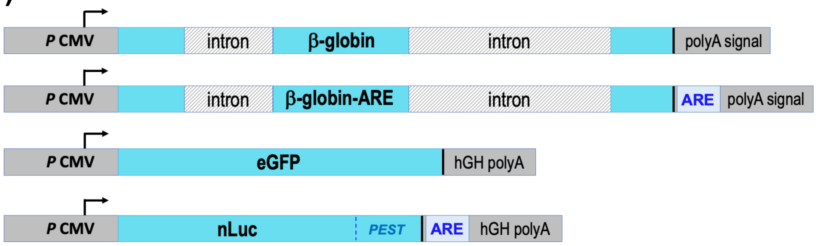

Supp Figure S6, Ranjan et al.

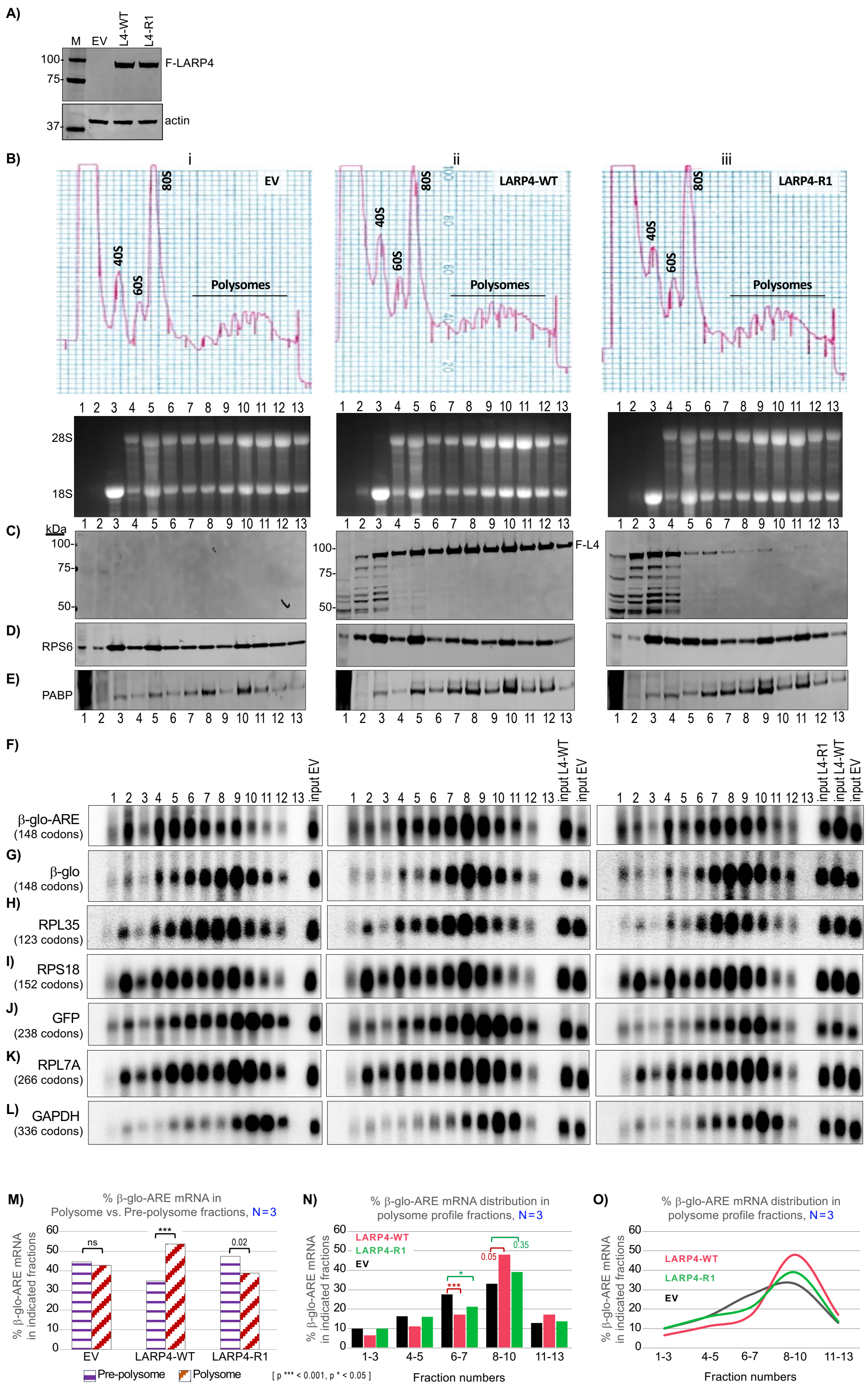

Supp Figure S7, Ranjan et al.

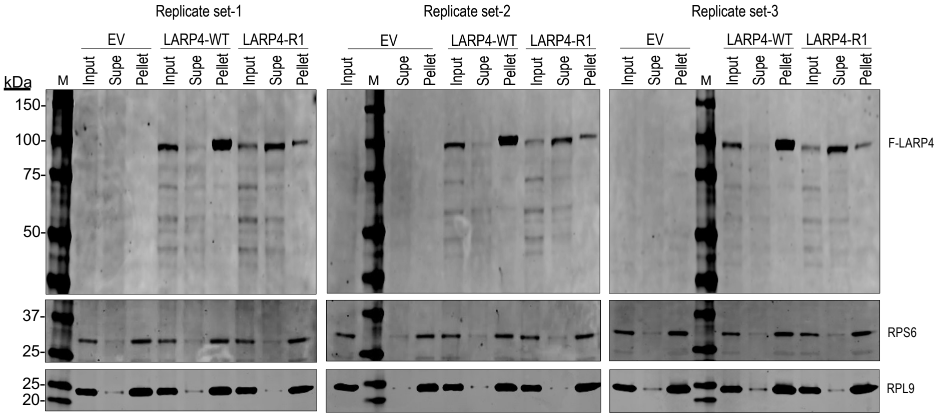

Supp Figure S8, Ranjan et al.

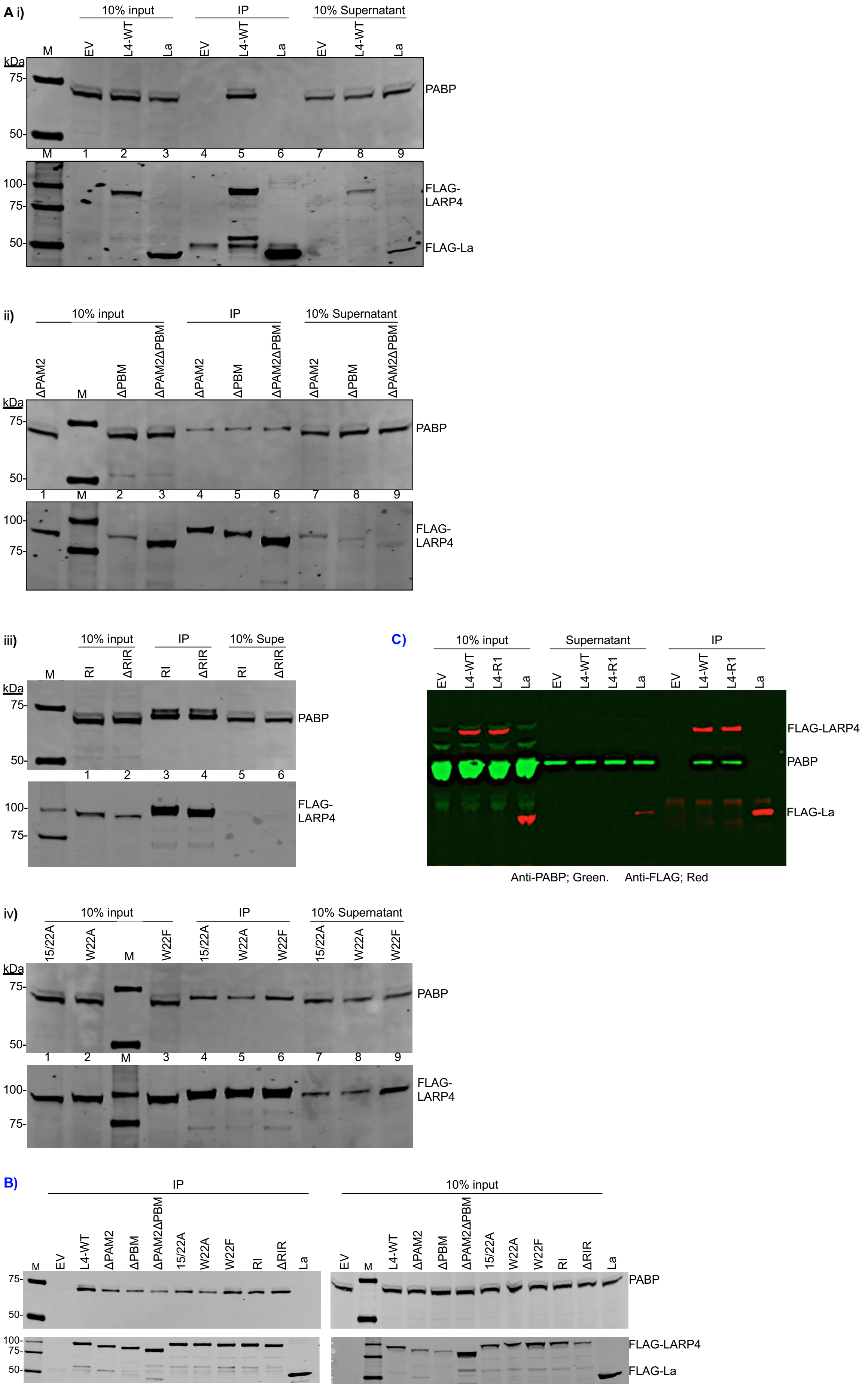

Supplementary Figure S9, Ranjan et al.

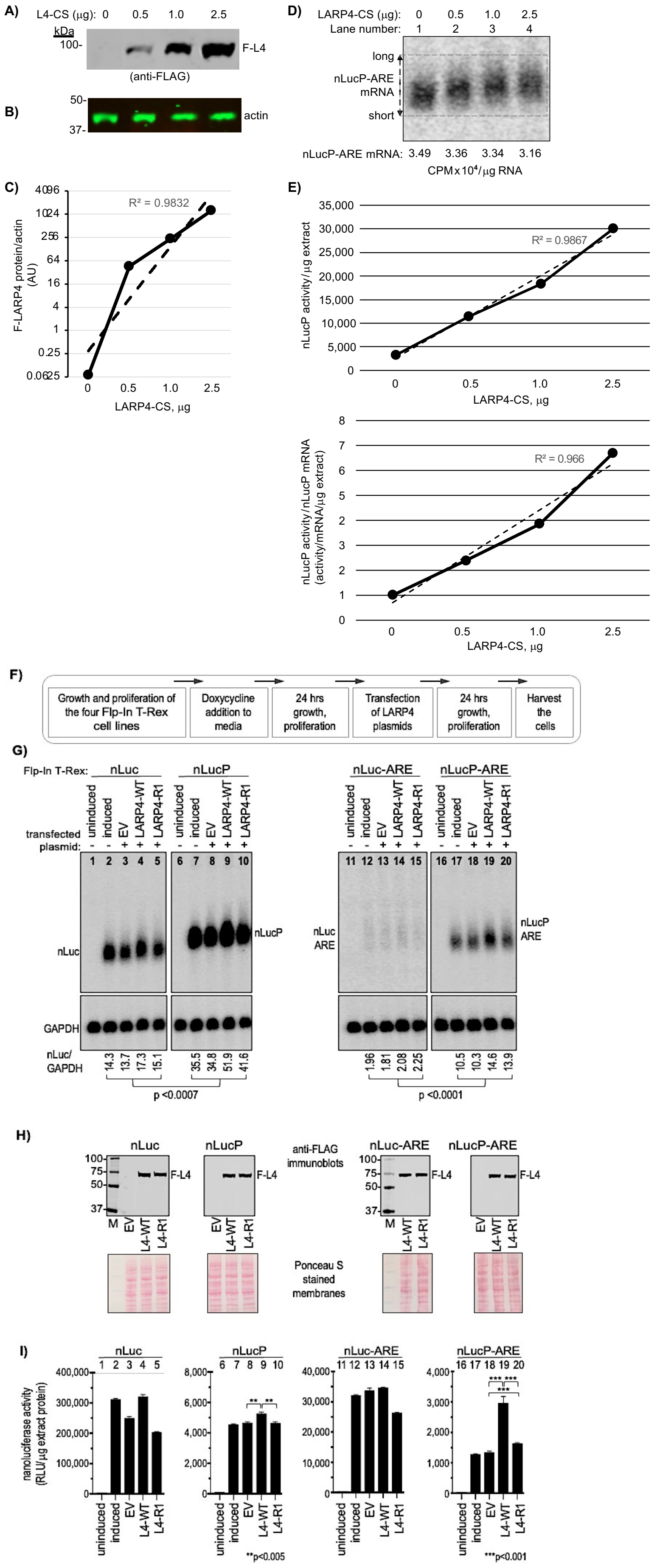

Supp Figure S10, Ranjan et al.

A) **Cryo-EM of 80S ribosome, modified/adapted from PDB:6Z6M**

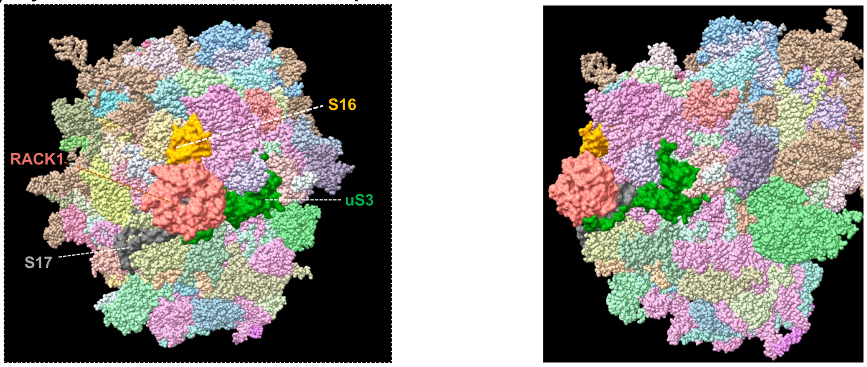

Cryo-EM of 80S ribosome, modified/adapted from PDB:6Z6M as above with other subunits blinded

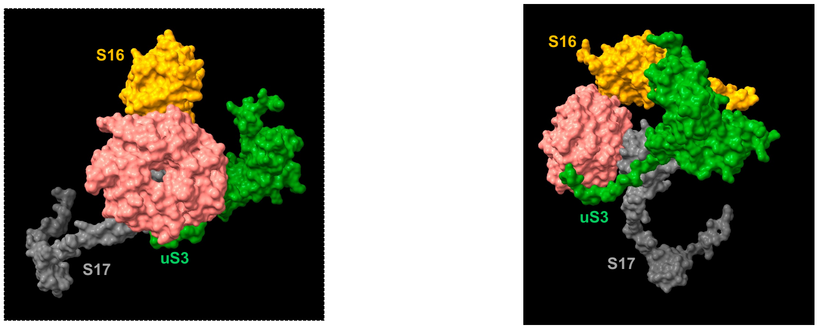

B) **Predicted by AlfaFold2-Multimer, assembled from RACK1 (salmon), ribosomal proteins uS3, S16 and S17 that interact with RACK1, and LARP4 CR1 and CR2 sequences (magenta).**

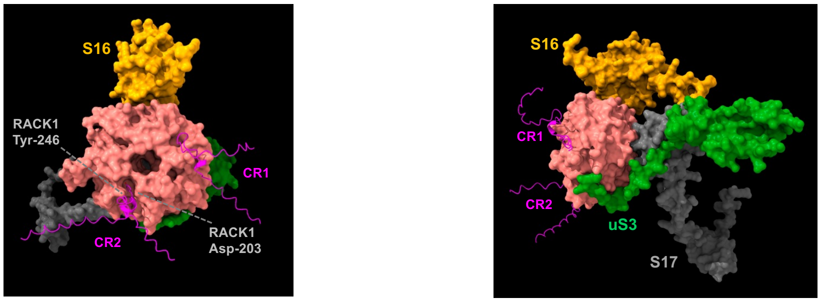

Supp Figure S11 Ranjan et al.

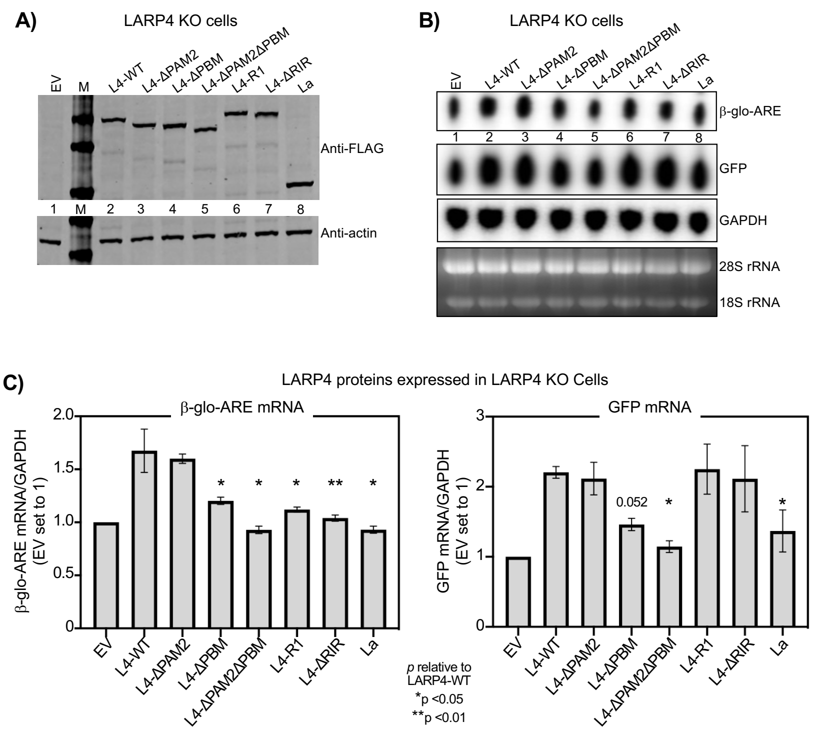

Supplement: Supplement 1 [file NIHPP2024.11.01.621267v1-supplement-1.pdf]
